# Residual Stress Evolution in Low-Alloyed Steel at Three Different Length Scales

**DOI:** 10.3390/ma16072568

**Published:** 2023-03-23

**Authors:** Silvia Leitner, Gerald Winter, Jürgen Klarner, Thomas Antretter, Werner Ecker

**Affiliations:** 1 Materials Center Leoben Forschung GmbH, Roseggerstraße 12, 8700 Leoben, Austria; silvia.leitner@mcl.at; 2 Voestalpine Tubulars GmbH & Co KG, Alpinestrasse 17, 8652 Kindberg-Aumühl, Austria; gerald.winter@vatubulars.com (G.W.); juergen.klarner@vatubulars.com (J.K.); 3 Institute of Mechanics, Montanuniversitaet Leoben, Franz Josef Strasse 18, 8700 Leoben, Austria; thomas.antretter@unileoben.ac.at

**Keywords:** finite element method, residual stresses, higher order stresses, phase transformation, heat treatment, low-alloyed steel

## Abstract

Quantitative and qualitative residual stress evolution in low-alloyed steel during heat treatment is investigated on three different length scales for sourgas resistant seamless steel tubes: on the component level, on the level of interdendritic segregation and on precipitate scale. The macroscopic temperature, phase and stress evolution on the component scale result from a continuum model of the heat treatment process. The strain and temperature evolution is transferred to a mesoscopic submodel, which resolves the locally varying chemistry being a result of interdendritic segregation. Within the segregation area and the surrounding matrix precipitates form. They are categorized with respect to their tendency for formation of microscopic residual stresses. After rapid cooling macroscopic stresses up to 700 MPa may form dependent on the cooling procedure. Mesoscopic stresses up to Δ50 MPa form depending on the extent of segregation. Carbides and inclusions occuring in low-alloyed steel are ranked by their tendency for residual stress formation in the iron matrix. This scale bridging study gives an overview of residual stresses, their magnitude and evolution on three different length scales in low-alloyed steels and the results presented can serve as a input for steel design.

## 1. Introduction

This work discusses the residual stress evolution in low-alloyed steel on three different length scales by the example of a seamless steel tube exposed to a quenching and tempering procedure. The residual stresses in low-alloyed steels are of interest, since they may deteriorate the steel’s mechanical performance. They can affect fatigue behavior [1] and also influence the steel’s interaction with hydrogen [2]. Especially in high-strength sourgas resistant steels, the interplay between residual stresses, corrosion and hydrogen susceptibility is an essential aspect of material development, as for example illustrated in [3]. This steel class is also an ideal candidate for future hydrogen transport and storage applications, where quantifying residual stresses and understanding their formation mechanisms is important to guarantee component integrity. Since residual stresses exist on different length scales, i.e., they self-equilibrate at characteristic lengths [4], their scale and magnitude which determine the mechanical performance are not known a priori. Bouchard and Withers displayed which scales are to be considered by the example of a weld line in steel in 2004 [4]. They classify type I as macroscopic residual stresses, where the characteristic length λ over which they self-equilibrate is about the component size S; type II residual stresses appear on length scales of 3g≤λ≤10g, where *g* denotes the grain size; for type III residual stress λ≤g. In this work we refer to type I stresses as macroscopic, type II as mesoscopic, and type III as microscopic residual stresses.

Modeling approaches to combine different length scales were, for example, published by Rammerstorfer et al. [5] for a high speed tool steel. Golanski [6] published in 1997 a general approach on how to homogenize and transfer the effect of residual stresses from one scale to another.

The outline of this work is to present a scale bridging investigation of residual stress evolution in low-alloyed steels by the example of a component, i.e., a tube. This multiscale approach includes the residual stress evolution on the component level, the interdendritic segregation line level and on precipitate level, thus addressing three relevant characteristic length scales in steels. The macroscopic residual stress state is relevant for component design and possible effects on the load bearing capacity or the components interaction with hydrogen [2]. The mesoscopic residual stress state could be of interest for damage mechanisms where crack formation occurs along segregation lines, such as typical for hydrogen induced cracking (HIC) [7]. Residual stress fields around precipitates may be of interest for example with respect to hydrogen trapping [8]. A thorough understanding of the underlying formation mechanisms and their magnitudes allows a better in-depth understanding if- and to what extent- the residual stresses affect the components behavior on a certain length scale.

The multiscale stress evolution is presented by the example of a seamless steel tube exposed to a quenching and tempering procedure. This allows to understand their evolution throughout a typical manufacturing process step.

The macroscopic model is briefly introduced and linked with a mesoscopic and a microscopic submodel to investigate the higher order stresses. The stress evolution on the macroscopic scale is experimentally verified and discussed in detail in [9,10]. The now presented work focuses on the multiscale modelling taking the macroscopic results as starting point.

To clearly define the macroscopic, mesoscopic and microscopic scale, first their length scales and characteristics are outlined.

### 1.1. Macroscopic Residual Stresses

When modeling residual stresses, one should keep in mind that they are never directly experimentally accessible for comparison, but are always calculated from residual strains. A correct assumption for the elastic properties is therefore essential and will be a main part of this work for evaluation of the higher order stresses. The macroscopic stresses are accessible via strain measurement techniques, such as technological experiments with strain gauges or cut compliance tests [11]. A combined application of these methods to determine the residual stress state in a welded joint is presented in [12]. The stresses are then calculated from strains using elastic, macroscopic material properties. The final stress state can be characterized this way after any manufacturing step. An accompanying thermo-mechanical simulation accounting for the phase transformations provides insight to the stress formation mechanisms during processing.

For the presented example of the seamless low-alloyed steel tube the residual stresses on the macroscopic scale are calculated using a continuum model developed in [9]. This computations provide phase fraction and temperature evolution as a function of the tube’s radial position during heat treatment consisting of quenching and optional subsequent tempering. A study on how to adapt the quenching process in order to lower residual stresses is published in [10]. The model and the results are briefly introduced in this paper and linked to the stress evolution on lower length scales.

### 1.2. Mesoscopic Residual Stresses

The mesoscopic model of a section of the tube’s wall focuses on residual stresses around segregation lines (planes), caused by interdendritic segregation during casting. This segregation produces local chemical inhomogeneities which can be the source of residual stresses.

The mesoscopic residual stresses (strains) around segregated areas referred to in this work, are difficult to access experimentally. They equilibrate in steels on length scales which are on an intermediate level between the grain structure on the lower boundary and the macroscopic continuum properties on the upper bound (1g≤λ≤3g), and are on a slightly smaller scale than in the definition given by [4]. Due to the chemical inhomogeneities, properties such as lattice parameters and stiffness change locally, which complicates the correlation of strain fields with stress fields.

Siwecki and colleagues [13] calculated the mesoscopic residual stresses due to segregation in samples cut from heavy steel plates after quenching. They analysed the local composition and observed chemical variations for Cr and Mo in segergated and depleted zones which are comparable to this work, but they observed larger changes in local carbon content. A simplified model of a segregation line is used in this work, where the chemical variations are accounted for, since they affect phase transformation behavior, the concomitant volume expansion, thermal expansion, and stiffness. The effect of transformation induced plasticity (TRIP) on lower length scales can be thus investigated.

An experimentally verified macroscopic phase transformation model is first transferred to the mesoscopic scale considering the segregated and depleted zone as two continua with different properties. The transformation kinetics, as well as relevant elastic, plastic and thermo-physical material properties are varied for the two different zones with respect to chemical composition. Thus the effect of a composition change on residual stresses can be studied.

The following assumptions were made to account for the local composition change:

- The content of Cr, Mo and C vary between the segregated and depleted region. This variation affects the martensite start temperature, thermal expansion, volumetric change during phase transformation and the Young’s modulus in the different regions. They are adjusted accordingly. For carbon only the relative composition change is known from high energy XRD measurements and not the absolute content. This complicates the estimation of consistent elastic parameters on lower length scales and made an iteration step prior to the residual stress calculations necessary.
- The local chemical variation may also affect the number, shape and size of precipitates. However, this effect is neglected in this work and shape and size of precipitates is considered to be the same in both segregated and depleted regions. Also the effects of precipitates on plastic properties are neglected, as only the elastic regime is relevant for residual stress formation. In the elastic regime, the properties of matrix and precipitates are homogenized using the microscopic model.

### 1.3. Microscopic Residual Stresses

The microscopic substructure is implemented by means of a “characteristic”’ volume element taking precipitate stress fields into account with prolate to oblate ellipsoids with an experimentally verified phase fraction randomly distributed within the matrix.

Microscopic stresses around inclusions or even around dislocation cores can experimentally be accessed with high resolution imaging techniques, such as TEM or high-resolution TEM [14] or also using X-ray diffraction and can again be calculated from strain maps [11].

A modeling approach was selected to determine microscopic stresses in low-alloyed steel, to give a qualitative and quantitative ranking of different inclusion types, such as oxides, nitrides and carbides, as well as to determine average and local effects of inclusions on the matrix stress state. First the average residual stresses in matrix and inclusion is calculated based on thermo-elastic considerations after [15] for common inclusion types in order to rank them by their tendency for stress formation. Then the histograms for maximum principal stresses as a function of precipitate volume fraction are compared.

## 2. Models, Materials and Methods

To discuss the residual stress evolution qualitatively and *quantitatively* discussed on three different length scales a self consistent multiscale approach is necessary. The following sections describe the employed scale bridging procedure that was used to determine the elastic constants for self-consistent multiscale model. Then the manufacturing process and the macroscopic model is briefly introduced. And subsequently the mesoscopic and microscopic models are presented.

### 2.1. Scale Bridging

The homogenization and scale bridging performed in this work are two steps to set up a self consistent multiscale model. First the elastic properties on the different length scales must be determined in an iterative procedure. After determining the elastic properties, the residual stresses can be investigated.

**Figure 1:**
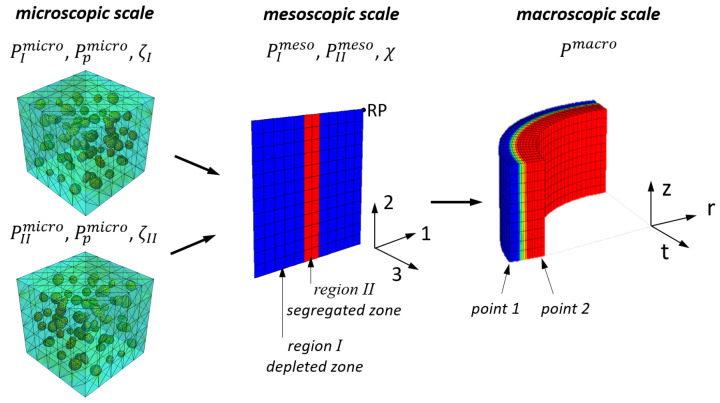
Schematic overview over the models and their properties $P_{i}^{micro}$, $P_{i}^{meso}$ and $P_{i}^{macro}$ on the different length scales.

The models used to describe residual stresses on three different length scales are shematically depicted in Figure 1: (i) a macroscopic process model calculating the temperature, phase and residual stress evolution, developed in [9,10] (ii) a model on the mesoscopic scale describing an infinite segregation “line” (plane) and (iii) a volume element containing inclusions for calculating microscopic stresses. In the microscopic model the material properties of matrix $P_{i}^{micro}$ are mixed with the precipitate properties $P_{p}^{micro}$ of the volume fraction $\zeta_{i}$ for the regions i=I,II to obtain the mesoscopic properties $E_{I}^{meso}$ and $P_{II}^{meso}$. In the mesoscopic model $P_{I}^{meso}$ and $P_{II}^{meso}$ are mixed with the experimentally determined volume fraction χ to calculate the macroscopic property $P^{macro}$. For two different points point 1 and point 2 the mesoscopic residual stresses are calculated by means of a mechanical submodel using the reference point RP. The microscopic residual stresses are calculated using $P_{i}^{micro}$ and $P_{p}^{micro}$ for different precpitate volume fractions $\zeta_{i}$.

On the mesoscopic scale two different model variants are used: an *unconstrained* model, were the reference point (RP, see Figure 1) remains unconstrained during cooling in order to isolate the contribution of chemical variation on residual stress formation. In the second mesoscopic model, referred to as the mesoscopic *submodel*, the thermal and mechanical boundary conditions from different points of the marcoscopic model (i.e., on the outer surface point 1, and on the inner surface point 2) are transferred to this model in order to determine the effect of macroscopic gradients on the mesoscopic scale in addition to chemical variation. Since the mesoscopic model describes a selected volume element and not a representative volume element (RVE), the applied boundary conditions are compensated for the elastic anisotropy due to the geometrical constellation in the selected volume element.

On the microscopic scale, it is assumed that the occuring precipitates form during tempering and are not present when the phase transformations happen. So it is safe to assumed that the microscopic stresses appear superimposed to the macroscopic and mesoscopic stresses and no constrained submodel connetecd to the macroscopic model is necessary here. Only an unconstrained model is used in this case to investigate the magnitude of residual stresses.

In the mesoscopic model the direction 3 coincides with the tangential direction *t*, and direction 1 with the radial direction *r* of the macroscopic model. For the microscopic model, the precipitates are statistically oriented, resulting in an isotropic material response of the volume element and no sub-coordinate system is given here.

The mesoscopic residual stress formation is influenced by local chemical inhomogeneities, which alter phase transformation kinetics and material properties. The considered local property changes *P* are the thermal expansion α, the metallurgical volume expansion ΔV and the Young’s modulus $E_{I}^{micro}$ and $E_{II}^{micro}$ for the depleted region (region I, see Figure 1) and the segregated region (region II, see Figure 1).

The depleted region I and the segregated region II can differ in precipitate volume fraction $\zeta_{i}$, for i=I,II. Thus the mesoscopic Young’s modulus $E_{i}^{meso}$ depends on the contribution from precipitates $E_{p}^{micro}$ and the matrix contribution $E_{i}^{micro}$, which is dependent on the carbon content. In the presented case it is assumed that the regions I and II contain the same type and volume fraction of precipitates.

The mesoscopic moduli $E_{i}^{meso}$ for the regions i=I,II can be calculated using a homogenization function *f*:

(1) $$E_{i}^{meso}=f\left(E_{i}^{micro},\zeta_{i},E_{p}^{micro}\right)$$

The homogenization function *f* can range from the Voigt upper bound of ($\left(1-\zeta_{i}\right)E_{i}^{micro}+\zeta_{i}E_{p}^{micro}$) to the Reuss lower bound ($\left(1-\zeta_{i}\right)/E_{i}^{micro}+{\left(\zeta_{i}/E_{p}^{micro}\right)}^{-1}$). For the investigated case *f* is between the upper and lower bound and is calculated numerically using the microscopic model. The matrix moduli $E_{I}^{micro}$, $E_{II}^{micro}$ are necessery input parameters and should be adjusted in region I and region II based on the carbon content, see [16]. However, the total carbon content in region I and II is unknown and only the variation in carbon content can be experimentally determined using XRD. This complicates the scale bridging procedure, since only the difference bewteen $E_{I}^{meso}$ and $E_{II}^{meso}$ is fixed by the difference in carbon content, but not their absolute values.

**Figure 2:**
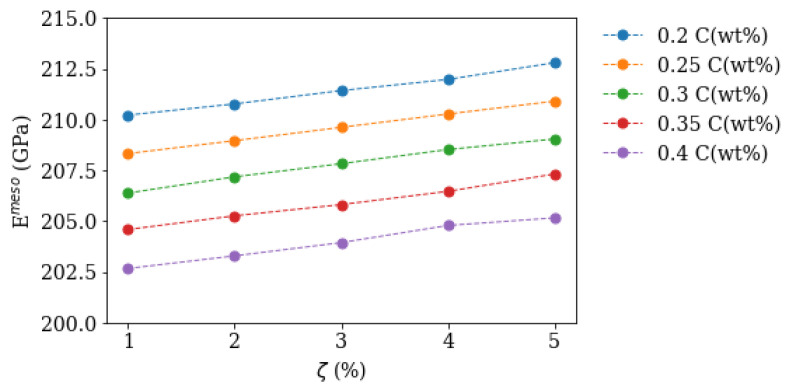
Calculated Young’s modulus $E^{meso}$ as a function of precipitate volume fraction ζ and different matrix carbon contents using the microscopic model as homogenisation function *f*.

Therefore the mesoscopic modulus $E^{meso}$ is first calculated as a function of precipitate volume fraction ζ and carbon content using the microscopic model as homogenisation function *f*. This data serves as a preliminary result and is shown in Figure 2.

When the precipitate volume fraction ζ and segregation line fraction χ are known from experiment, the mesoscopic $E_{i}^{meso}$ moduli for region *i = I, II* can be combined using the unconstrained mesoscopic model to calculate the macroscopic modul $E^{macro}$, which must match macroscopically determined tensile test data.

(2) $$E^{macro}=F\left(\chi ,E_{I}^{meso},E_{II}^{meso}\right).$$

In the elastic consideration, the stiffness of the embedded precipitates adds to these moduli in the depleted region I and the segregated region II.

With the thus determined microscopic and mesoscopic elastic properties, the stresses on the corresponding length scales can be calculated with the above presented finite element models for quenched and tempered state. To quantify the microscopic residual stresses around inclusions and precipitates, a number of common inclusions in low-alloyed steel is selected and compared with respect to their potential for thermo-elastic residual stress formation.

### 2.2. Phase Transformation Model

The phase transformation model describes the displacive phase transformations for two product phases $z_{i}$ for i=1,2 from austenite to martensite and bainite.

The kinetics is governed by the Koistinen Marburger kinetic [17] for transformation to martensite and by a model developed by Garrett and Mahnken [18,19] for transformation to bainite. The model parameters were adjusted using dilatometer curves for different cooling rates and can be found in our previous publication [9]. The phase transformation kinetic is combined with the concomitant changes in material properties and transformation related effects by a user defined field in ABAQUS [20]. The current phase composition is assigned to field variables and the temperature dependent data for elasticity, yield curves, thermal expansion, thermal conductivity and specific heat of the individual phases are mixed accordingly [9,10].

The phase transformation from austenite to martensite primarily depends on the carbon content, which alters the martensite start temperature. An exponential correlation model for change in martensite start temperature [21] as a function of local composition is implemented. The bainite phase transformation also depends on carbon and other alloying elements, but for the work at hand was considered to be unchanged since under process conditions no bainite is formed.

### 2.3. Macroscopic Model

The macroscopic phase transformation model was developed to calculate the temperature, phase and residual stress evolution in seamless steel tubes during accelerated cooling [9,10]. In the investigated manufacturing setup, the tube moves trough a cooling equipment consisting of eight cooling baskets, which apply spray water. This yields a non-continuous cooling progress. Temperature gradients, phase evolution and residual stresses differ on the inner and outer surface. The temperature dependent elastic, plastic and thermophysical properties for the macroscopic model are used from [9,10].

The macroscopic cooling process is investigated for a tube of 12.65 mm wall thickness and 177.8 mm outer diameter. The non-continuous cooling on the outer surface and high temperature gradients yield a “spiky” temperature profile over time, while on the inside the temperature decays continuously over time. Two submodels are derived form this macroscopic model: (i) on the outer and (ii) on the inner surface. The effect of different local temperature evolution and the tube’s macroscopic mechanics on segregation lines is investigated.

### 2.4. Mesoscopic Model

In the mesoscopic model (see Figure 1), residual stresses in an infinite segregation plane (called segregation “line”) and the depleted areas are investigated.

**Figure 3:**
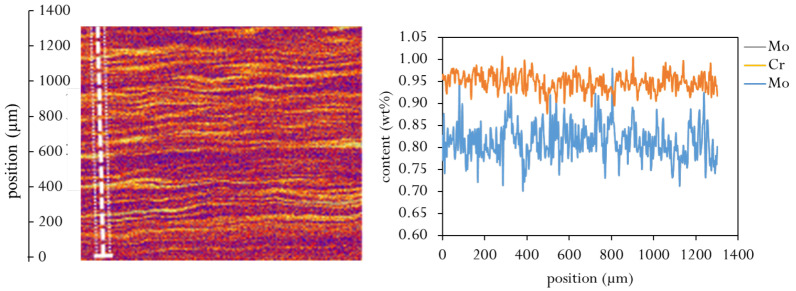
Segregation profile of Mo and Cr across a line scan (white line) detected using electron probe micro analysis (EPMA).

Since the chemical composition in the segregation lines differ from the depleted zones in Mo, Cr and C concentration. Residual stress evolution is affected by a change in mechanical, thermo-physical and phase transformation properties. The local chemical enrichment in the segregation lines was determined using electron probe micro analysis (EMPA) and wavelength dispersive X-ray analyses (WDS) with a scan size 3.5×3.5 μm^2^, 15 kV and 40 ms dwell time over the tubes cross section and an exemplary measurement is shown in Figure 3. The local enrichment in carbon was calculated from high-energy X-ray diffraction on 2 mm thick tube segments, where macroscopic tangential, axial and radial stresses are relieved. The measurements were performed at the P07 beamline of Helmholtz-Zentrum Geesthacht at PETRA III synchrotron source of DESY in Hamburg with an energy of 87 keV. From the determined lattice parameter in the as-quenched state, the relative carbon variation was calculated using the correlation published by Moyer [22]. A maximum to minimum difference of 0.03 wt%C was determined. For the calculations, a much broader change in carbon content is considered to evaluate a broader spread of chemical variation.

As mentioned before, two submodels on the tube’s outer (point 1) and inner (point 2) surface are investigated. The mechanisms driving this mesoscopic residual stress formation are revealed using an *unconstrained* mesoscopic model without the submodel boundary conditions from the macroscopic model that is subjected to cooling. The boundary conditions in the unconstrained case are generalized plane strain boundary conditions in the three directions 1, 2, 3 since the model is small compare to the tube’s wall thickness. It is later shown, that this simplification is possible since the mesoscopic stresses act superimposed to the macroscopic stresses despite all non-linearities during phase transformation.

**Figure 4:**
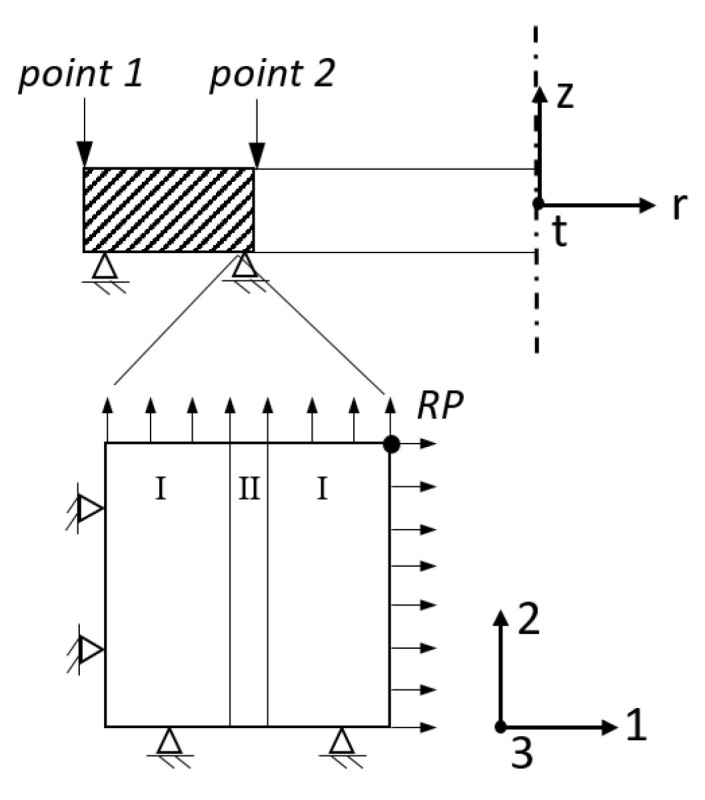
Schematic model description of the macroscopic axisymmetric process model with constant strain in axial direction ($\varepsilon_{z}$ = const.) and the submodel on the outer (point 1) and inner (point 2) tube’s surface as generalized plane strain model in direction 1, 2 and 3. In the submodel contains depleted regions (I) and segregated regions (II). The macroscopic boundary conditions are transferred via the reference point RP.

For the *submodeling* case the displacements resulting from the macroscopic model are transferred to the reference point while the outer faces preserve a generalized plane strain state, as shown in Figure 4.

The mechanical properties, including the elastic and plastic material properties mainly depend on grain size and local carbon content. Bechet-Beaujard etching and electron back scattering diffraction (EBSD) pattern revealed, that the former austenite grain size and martensite structure are unaffected by the chemical variation. Thus, it is assumed that the plastic and elastic properties in austenite are equal for segregated and non-segregated areas. The occurring stresses after phase transformation do not exceed the product yield strength in martensite and thus only the change in elastic properties of the product phase was considered relevant. Souissi [16] has calculated the dependence of Young’s modulus in martensite as a function of carbon content and with the determined carbon variation, the moduli vary by 0.5% between segregated and non-segregated area.

**Figure 5:**
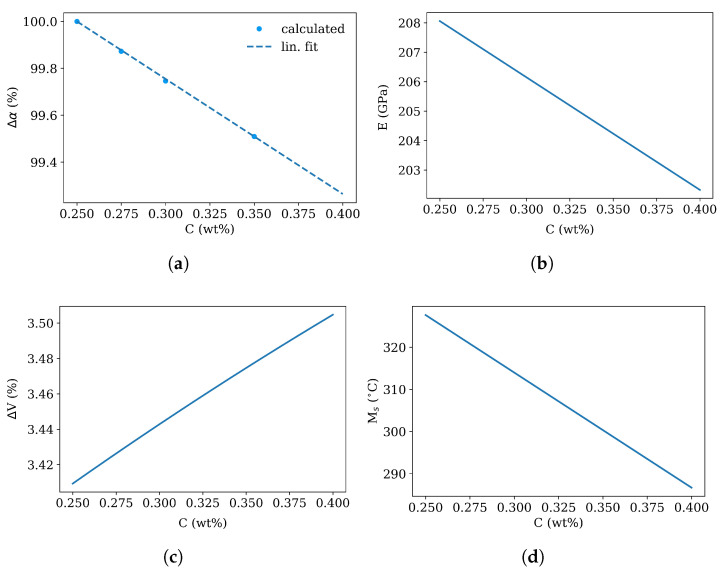
Dependency of difference in thermal expansion coefficient Δα (JMatPro, [23]) and metallurgic volume expansion ΔV ([22]) as function of carbon content as well as Young’s modulus *E* ([16]) and martensite start temperature $M_{s}$ ([21]) as function of carbon content.

**Figure 6:**
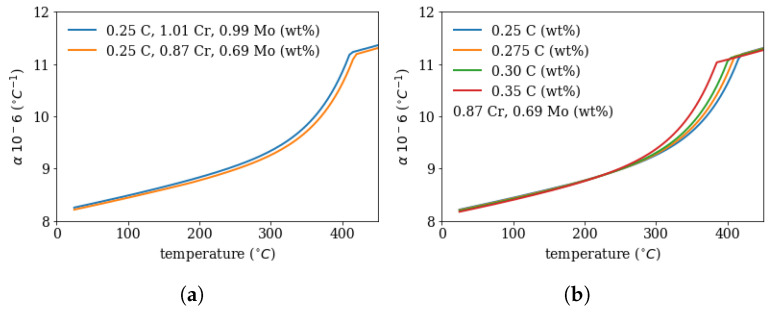
Calculated thermal expansion coefficient α (with JMatPro) as function of temperature for different (**a**) Cr and Mo content for the depleted region I (0.87%Cr, 0.69%Mo) and for the segregated region II (1.01%Cr, 0.99%Mo) and (**b**) carbon content in the depleted zone.

The phase transformation kinetics in the segregated (region II) and in the depleted region (region I) are derived from the macroscopic model with the following changes in the segregation line (region II):

- The thermal expansion is treated as a function of carbon content from 0.25 to 0.4 wt%. The plot in Figure 5a shows the JMatPro calculations for 0.25, 0.275, 0.3 and 0.35 wt%C avaraged over a temperature range from 25 °C to 370 °C with 0.25 wt%C as reference value, see Figure 5a. The full data set for the chemistry and temperature dependent thermal expansion is shown in Figure 6.
- The Young’s modulus in the segregated area is changed according to the carbon content, see Figure 5b following [16].
- The volume expansion is changed as a function of carbon content from 0.25 to 0.4 wt% using the work of [22], see Figure 5c.
- The martensite start temperature $M_{s}$ is shifted to lower temperatures due to the higher carbon, Mo and Cr content following the exponential model [21], see Figure 5d.

The total coefficient of thermal expansion was calculated using JMatPro [23] as a function of chemistry and is shown in Figure 6—for different carbon content an different Cr and Mo concentrations, which are the maximum and minimum values from Figure 3. The thermal expansion coefficient with the segregation chemistry is higher than for the depleted region. This implies, that during cooling without phase transformation the segregation line would contract more than the depleted zone and would be in the tensile stress regime.

In the unconstrained version of the mesoscopic model the thermal expansion, volume expansion, transformation start temperature and $E^{micro}$ are continuously varied as function of carbon content from 0.25 to 0.4 wt%C. For the submodel case, a carbon segregation of 0.03 wt%C is assumed. This yields a total carbon content in region I of 0.235 wt% and in region II 0.265 wt% for a precipitate volume fraction of ζ=4% and corresponds to a shift in $M_{s}$ by 13 °C between region I and II.

The length of the modeled region is 150 μm and the thickness of a characteristic segregation line ranges from 10–80 μm depending on the volume fraction and region, which is about 1–2 times the grain size *g*. A segregation plane thickness of 50 μm was modeled, which corresponds to a volume fraction χ of 20%. First the thermal and subsequently the mechanical problem is solved using quadratic brick elements DC3D20 and C3D20 respectively.

### 2.5. Microscopic Model

As the residual stresses around inclusions can on the one hand be locally very high, but self-equilibrate within small distances, and on the other hand may also increase the overall matrix stress depending on the volume fraction, a twofold approach was chosen. The average stress caused in the matrix is compared for different inclusion types, and then the effect of volume fraction on the stress distribution is investigated by means of a histogram for the maximum principal stress.

A matrix cube with embedded inclusions of varying shape and aspect ratio serves as statistical RVE to calculate the residual stresses and the Young’s modulus for different matrix carbon contents and precipitate volume fractions. The model is meshed with tetrahedral C3D10 elements of quadratic shape functions with a coarse element size of 0.2 μm on the outer surface compared to the cubes length of 1 μm, and strong element refinement near the inclusions. The inclusions have a main axis length ranging from 40–70 nm. This coresponds to precipitate sizes determined in the investigated example material using TEM and EBSD. The shapes could not be determined for all precipitate types and as a simplification all inclusions are modelled as ellipsoids with randomly varying aspect ratios from 0.5–2. They are meshed with an element size of maximum 10 nm. Periodic boundary conditions are applied the free surfaces. First, (i) a uniaxial displacement is applied on the reference node to determine the Young’s modulus $E^{meso}$ for different carbon content and precipitate volume fraction ζ and (ii) to determine the thermo-elastic residual stresses the reference point (RP) is unconstrained.

The microscopic residual stress formation as well as the ranking of different precipitate types is considered elastically below a temperature of 0.4 $T/T_{M}$ homologous temperature (about 400 °C) based on deformation mechanism maps in steel [24]. Above this temperature a stress free state is assumed and at cooling from 400 °C to 25 °C residual stresses start at a stress free state and form elastically. This thermo-elastic approach is a simplified measure and for a more precise analysis the crystallographic features and stress relaxation due to creep should be considered.

To calculate the thermo-elastic stresses a uniform temperature field is applied and the amplitude changed from a temperature of 400 °C to RT. A Young’s modulus $E_{I}^{micro}$ for a matrix carbon content of 0.25 wt%C with ν=0.33 and for the inclusions $E_{P}^{micro}=310$ GPa and ν=0.33 are assumed, which represents a mixture of Cr-rich cementite and Cr/Mo rich MC carbides, which have been shown to be relevant in similar alloys [25] and were also determined experimentally using TEM and EBSD for the investigated example material.

If a product is subsequently tempered, the macroscopic residual stresses fully relax at a temperature of 700 °C. During cooling to room temperature microscopic and mesoscopic residual stresses form due to thermal misfits between the steel and precipitates.

Isotropic elastic properties *E* and ν from literature are implemented as a starting value and the elastic properties for the precipitates are shown in Table 1 for representative carbides, nitrides and oxides. The thermal expansion and elastic data to determine residual stress formation potential of different precipitate types were mostly taken from first principle calculations and are- if available- average values from 400 °C to 25 °C. The resulting average stresses in matrix and inclusion were calculated following [15]. Assuming spherical inclusions in the depleted (i=I) and in the segregated region (i=II), the average stress $\bar{\sigma_{P}}$ can be calculated as follows:

(3) $${\bar{\sigma }}_{P}=\sigma_{11}=\sigma_{22}=\sigma_{33}=\frac{(\alpha_{i}-\alpha_{P})\Delta T}{\frac{1}{3K_{P}}+\frac{1}{4\left(1-f\right)G_{i}}+\frac{\zeta }{3\left(1-f\right)K_{i}}},$$

where *f* denotes the precipitate volume fraction, *K* the bulk modulus and *G* the shear modulus. The subscript *P* denotes the inclusion properties and i=I,II the matrix. The average matrix stress ${\bar{\sigma }}_{I,\;II}$ is

(4) $$\bar{\sigma_{i}}=\frac{-\zeta {\bar{\sigma }}_{P}}{1-\zeta }.$$

## 3. Results

To get an elastically consistent mesoscopic model and macroscopic model the Young’s moduli $E_{I}^{meso}$ and $E_{II}^{meso}$ ($E_{I,II}^{meso}$) were calculated to give a macroscopic modulus of 209 GPa [9] for a segregation line volume fraction χ=20% and precipitate volume fraction ζ=4% in both depleted and segregated region.

**Figure 7:**
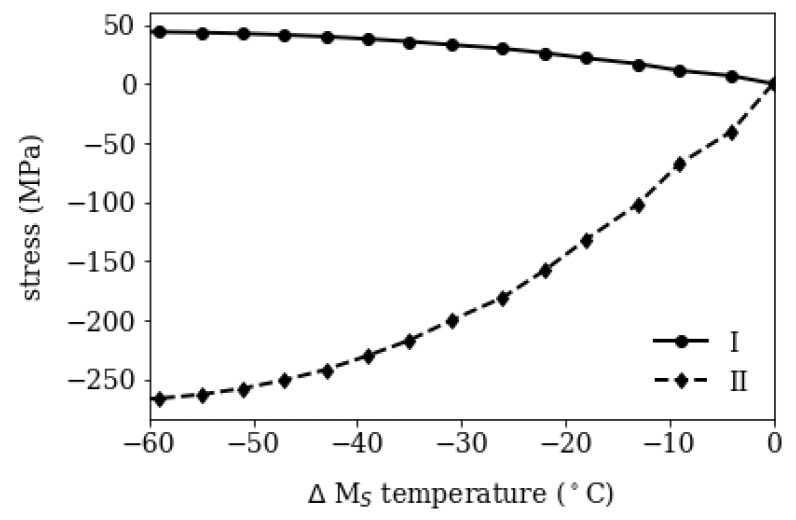
Residual stresses after cooling in depleted (I) and segregated (II) region for pure martensite as function of difference in martensite start temperature $\Delta M_{s}$.

In the unconstrained version of the mesoscopic model the martensite start temperature $M_{s}$, volume expansion ΔV and thermal expansion α are varied as a function of carbon content for a segregation line volume fraction χ=20%. The residual stresses are shown in Figure 7 over the local difference in $M_{s}$. In the case of pure martensite formation, the residual stresses increase with the difference in $M_{s}$ and saturate at a constant level as the difference increases. The depleted region (I) is in tensile stress regime, whereas the segregated region is compressed (II). This is a result from the TRIP effect and can be explained by imagining the stepwise transformation of the mesoscopic model depicted in Figure 1. The depleted region transforms into martensite first, due to its lower carbon content, and the concomitant volume expansion causes tensile stress and plastic deformation in the still austenitic segregated region (II). When the segregated region transforms with time/temperature delay, the additional plastic strain leads a compressive stress state after transformation.

**Figure 8:**
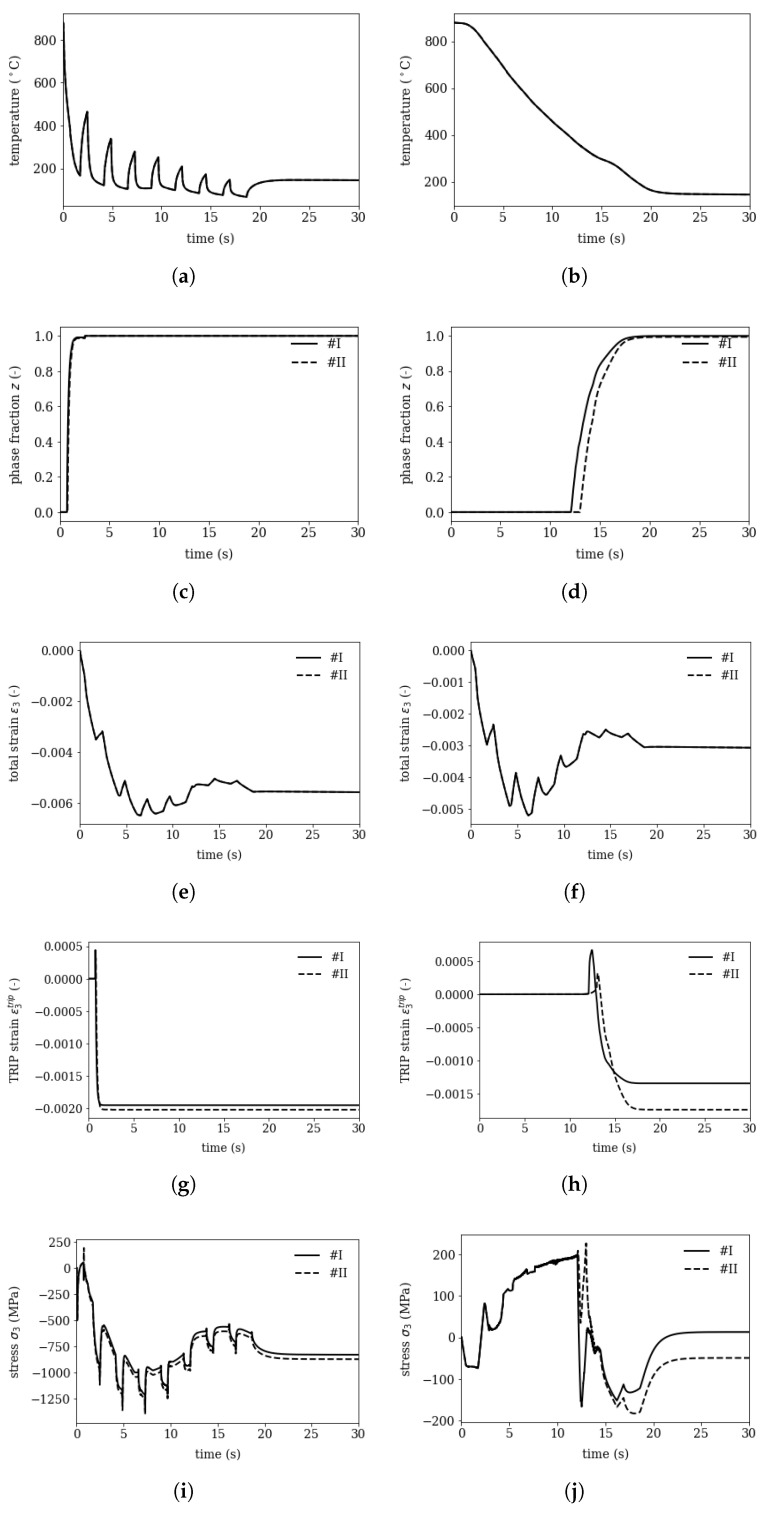
Results of mesoscopic submodel for (left) point 1 on the outer surface and (right) point 2 on the inner surface (see also in Figure 1): the temperature, phase fraction *z*, total strain in direction 3 (tangential direction in the macroscopic model), the TRIP strain and the resulting stress.

The mesoscopic submodel is subjected to the thermal and mechanical history and the results are shown in Figure 8 (left) on the outer surface and (right) on the inner surface. On the outer surface the temperature decreases stepwise due to the setup of the cooling baskets, while on the inner surface it decreases continuously. These results match exactly the profiles published in [9] in Figure 3.7a. Due to the lower martensite start temperature $M_{s}$ the transformation starts with a visible delay on the inner surface in the segregation line (region II).

The total strain $\varepsilon_{1}$, $\varepsilon_{2}$ and $\varepsilon_{3}$, where $\varepsilon_{3}$ corresponds to the macroscopic tangential strain, are applied with a reference node and are constant over the investigated mesoscopic model which is small compared to the tube’s wall thickness. The depicted trip strain $\varepsilon_{3}^{trip}$ represents the component of the transformation induced plasticity (TRIP) in direction 3. The TRIP strains $\varepsilon_{3}^{trip}$ on the inner and outer surface differ, since the stress states during transformation vary.

Comparing the residual stress evolutions $\sigma_{3}$ on the outer (point 1) and inner (point 2) surface reveal that the outer surface reaches residual stresses of −831 MPa (I) to −873 MPa (II) in tangential direction and the inner surface 13 MPa (I) to −50 MPa (II), where (II) refers to the segregated area and (I) to the depleted area.

The absolute level of mesoscopic residual stresses matches the findings from the macroscopic calculations and measurements published in [9]-since the elastic values are consistently chosen. The difference between the minimum and maximum values on the outer surface of 42 MPa and 63 MPa on the inner surface match with the values in Figure 8. This shows, that despite huge non-linearites during cooling and phase transformation the mesoscopic residual stresses are superimposed to the macroscopic stresses.

**Figure 9:**
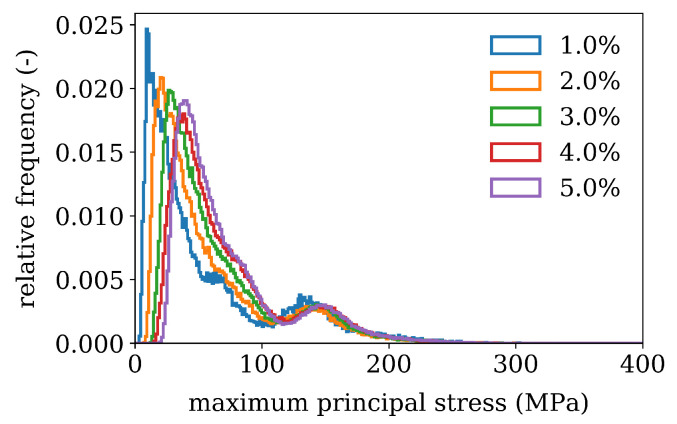
Histogram showing the relative frequency of maximum principle thermo-elastic stress after cooling for different precipitate volume contents ζ.

The relative stress formation potential (i.e., the average matrix stress $\sigma_{m,avg}$ of different inclusion types is given for an arbitrary volume fraction ζ of 4%, to get a relative comparison between the species in Figure 9. Boron nitride BN shows the highest residual stress formation potential in steel based on thermo-elastic assumptions, followed by ${\mathrm{Mo}}_{2}$C, TiC, NbC and VC. Cementite ${\mathrm{Fe}}_{3}$C causes low residual stresses compared to the other precipitates. MgO would theoretically cause negative residual stresses, due to its larger thermal expansion compared to bcc Fe. However, depending on the interface properties of MgO and the Fe matrix, the negative residual stresses could lead to void formation.

**Figure 10:**
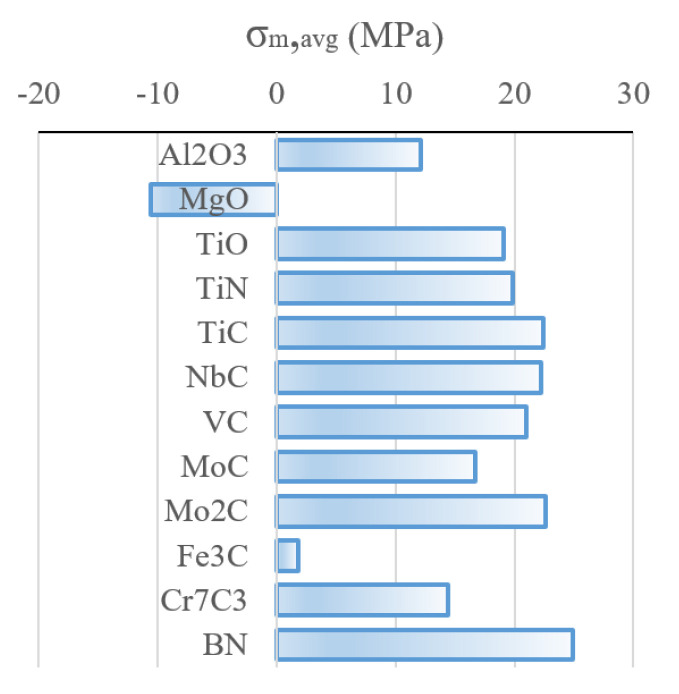
Residual stress formation potential in matrix for a precipitate volume fraction of ζ=4% for relative comparison different inclusion types, after [15].

For Cr-rich inclusions the maximum principle stresses of RVE’s with varying precipitate volume fractions ζ are evaluated at all integration points of the finite element model and weighted by the integration point volume. The results are shown in Figure 10. and show a bimodal distribution. The first peak, is the most frequently occurring stress changing from 15 MPa to 75 MPa for volume fractions from 1% to 5%. It is of similar magnitude as the calculated average matrix stress in Figure 9. The second peak is close to 150 MPa which occurs in the inclusion vicinity. The maximum values of range up to 550 MPa- they are mesh size dependent however, but similar mesh size was used to give a relative estimation.

**Figure 11:**
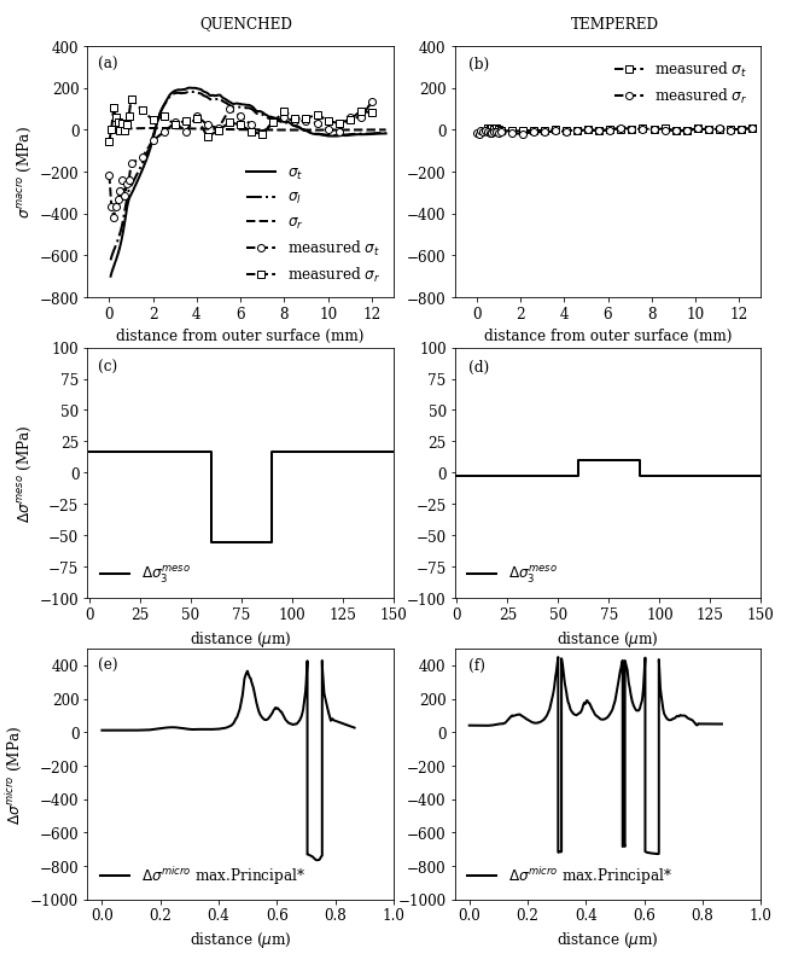
Macroscopic, mesoscopic and microscopic residual stresses for quenched (left) and tempered state (right).

Figure 11 shows a summary of residual stress formation on macroscopic, mesoscopic and microscopic scale for quenched/ or accelerated cooling and tempered condition. Macroscopic stresses form during cooling due to plastic thermal misfits and phase transformation and are fully released after tempering at about 700 °C, as has been experimentally shown in [40].

After quenching the stesses in the mesoscopic submodel in tangential direction of the tube (submodel direction 3, see Figure 1) around a single segregation line are ≤±75 MPa. The depleted region I is in tensile regime, and the segregation line (region II) is in compressive stress regime.

After tempering, with a thermal mismatch between the segregated and depleted region, stresses of ≤±25 MPa remain. The mesoscopic stresses act over wider ranges, but are small in size. After tempering, the segregated region is in tensile stress regime, since the thermal expansion in this region is higher than in the depleted region (compare Figure 6).

The microscopic stresses are higher compared to the mesoscopic ones and equilibrate over small distances ≤0.5 μm, which is important since residual stresses only can act as crack opening force, if they act on a larger scale than the defect size is. The microscopic stresses depend on the precipitate volume fractions, which may increase during tempering and the occurring precipitate types. The depicted results show the maximum principal stress along a path cutting through the microscopic model with 1% precipitate volume fraction (left) and 5% volume fraction (right) and show the compressive stress state within the precipitates and tensile stresses in the matrix.

## 4. Summary and Conclusions

A study of residual stresses in a sourgas resistant low-alloyed steel is presented on three different length scales.

Starting from a macroscopic process model, two smaller length scales were investigated, namely a mesoscopic model considering a simplified form of an interdendritic segregation line and a microscopic cubic model with embedded inclusions. The macroscopic results are transferred to these two submodels to investigate the magnitudes of residual stresses on lower length scales and cross-scale coupling effects, i.e., the effect of non-linearities and chemical inhomogeneities, such as interdendritic segregation. The residual stresses were evaluated and discussed in both, quenched and tempered state.

During quenching, mesoscopic interdendritic segregation can retard martensitic transformation in segregated regions. This leads to compressive stresses in the segregated areas and tensile stresses in the depleted areas due to the TRIP effect. The study of two points, one at the outer (point 1) and one at the inner (point 2) surface of the tube, shows that the mesoscopic residual stresses superimpose the macroscopic stresses despite many nonlinearities (phase transformation, plasticity, TRIP, …). Thus, the mesoscopic model can be used independently of the macroscopic history to evaluate the effects of carbon content and composition.

Such a mechanically unconstrained, independent mesoscopic model is employed to investigate the residual stresses in segregated areas and depleted areas as function of carbon content. The residual stresses saturate after a certain difference in $M_{s}$ in the investigated 50 μm thick segregation line (representing a volume fraction of 20%) at a level of −270 MPa and in the depleted region at +50 MPa.

After tempering the mesoscopic residual stresses are not dominated by the transformation mechanism but by the difference in thermal expansion between segregated and depleted areas. This leads to tensile stresses in the segregated region after tempering, due to its higher coefficient of thermal expansion compared to the depleted region. The resulting residual stresses are small, i.e., ≤±25 MPa.

The microscopic residual stresses around precipitates, are investigated using both a cubical representative volume element (RVE) with embedded inclusions and an averaging approach. With the RVE, the maximum principal thermo-elastic residual stresses of inclusions are investigated for different volume fractions. The histogram of the stresses at integration points is discussed and the most frequent stress correlates with the calculated thermo-elastic average matrix stress and ranges from 10 to 70 MPa. A selection of inclusions is ranked by the average matrix stress, which they cause for a given arbitrary volume fraction. It turns out, that BN shows the highest relative residual stress formation potential, followed by ${\mathrm{Mo}}_{2}$C, TiC, NbC and VC. Only MgO stands out, due to its larger thermal expansion compared to bcc Fe it theoretically creates negative residual stresses in the matrix, which could lead to voids.

This multiscale investigation shows how stresses on three length scales develop. The impact of residual stresses on the material behavior (e.g., interaction with hydrogen, corrosion resistance, load bearing capacity, etc.) can be better assessed by this quantitative analysis and also opens the door to computational multiscale stress design.

## Figures and Tables

**Table 1:** Elastic and thermal properties and thermoelastic stresses for a number of precipitates at a volume fraction of 4% in an iron matrix.

	$\alpha^{a}$	B	E	G	ν	$\sigma_{p}$	$\sigma_{m}$
	$\left(10^{-6}K^{-1}\right)$	(GPa)	(GPa)	(GPa)	(-)	(MPa)	(MPa)
Al_2_O_3_	7.278 [26]	249 [27]	399 [27]	162 [27]	0.24 *	−402.5	21.2
MgO	1.272 [26]	164 [27]	310 [27]	130 [27]	0.19 *	351.0	−18.5
TiO	5.783 [28]	262 [29]	294 *	112 *	0.31 *	−633.1	33.3
TiN	5.678 [30]	277 *	459 [31]	188 [31]	0.22 [31]	−658.7	34.7
TiC	4.918 [30]	242 [32]	474 [32]	187 *	0.17 *	−746.1	39.3
NbC	5.101 [31]	267 [32]	438 [32]	188 *	0.23 *	−739.3	38.9
VC	5.532 [31]	308 [32]	513 [32]	210 *	0.22 *	−699.3	36.8
MoC	6.507 [32]	328 [32]	410 [32]	159 *	0.29 *	−554.8	29.2
Mo_2_C	5.167 [33]	300 [32]	322 [32]	122 *	0.32 *	−751.6	39.6
Fe_3_C	9.583 [34]	224 [35]	242 *	92 [35]	0.36 *	−59.8	3.1
Cr_7_C_3_	6.958 [36]	312 [37]	226 *	82 *	0.38 *	−477.5	25.1
BN	5.000 [38]	400 [39]	923 *	414 *	0.11 *	−828.7	43.6
Fe	10.00 [9]	174 [9]	209 *	80 *	0.3 [9]		

^a^ If available, average value from 700 to 25 °C. * Calculated from cited literature values in the given reference.

## Data Availability

Data sharing not applicable.
